# Propranolol, post-traumatic stress disorder, and intensive care: incorporating new advances in psychiatry into the ICU

**DOI:** 10.1186/s13054-014-0698-3

**Published:** 2014-12-19

**Authors:** Andrew John Gardner, John Griffiths

**Affiliations:** Faculty of Medicine, Oxford University, John Radcliffe Hospital, Headley Way, Oxford, OX3 9DU UK; Nuffield Department of Anaesthetics, John Radcliffe Hospital, Headley Way, Oxford, OX3 9DU UK

## Abstract

Post-traumatic stress disorder (PTSD) is a common complication of an ICU admission. Rarely is there a continuation of care, which is aimed at screening for and treating this debilitating disease. Current treatment options for PTSD are held back by inconsistent efficacy, poor evidence, and a lack of understanding of its psychopathology. Without ‘gold standard’ assessment techniques to diagnose PTSD after an ICU admission, the development of care pathways is hindered. This paper advocates for two interwoven advances in psychiatric care (specifically for PTSD) after ICU: (1) incorporate the monitoring and treating of psychiatric co-morbidities during extended patient follow-up, and (2) rapidly adopting the latest research to maximize its benefit. The discovery that memories were not fixed, but malleable to change, set off a sequence of experiments that have revolutionized the approach to treating PTSD. It is hoped that the phenomenon of reconsolidation can be exploited therapeutically. In the act of remembering and re-storing traumatic memories, propranolol can act to dissociate the state of sympathetic arousal from their recollection. Often, ICU patients have multiple physical co-morbidities that may be exacerbated, or their treatment disrupted, by such a pervasive psychological condition. The rapid uptake of new techniques, aimed at reducing PTSD after ICU admission, is necessary to maximize the quality of care given to patients. Increasingly, the realization that the role of intensive care specialists may extend beyond the ICU is changing clinical practice. As this field advances, intensivists and psychiatrists alike must collaborate by using the latest psychopharmacology to treat their patients and combat the psychological consequences of experiencing the extremes of physiological existence.

## Introduction

Post-traumatic stress disorder (PTSD) is triggered after experiencing one or more traumatic events. A debilitating disease, PTSD is a common complication of admission to ICUs. With a prevalence of 5% to 64% among patients discharged from the ICU, this figure rivals the chances of developing PTSD after surviving cancer (1.9% to 39%) and a terrorist attack (30% to 40%) [1-3]. Current treatment options are held back by inconsistent efficacy, poor evidence, and a lack of understanding of its psychopathology. To address this, the link between the formation of memories, their maintenance, and how they relate to the symptomatology of PTSD has been studied. As part of a new psychopharmacological approach to treating PTSD, drugs such as propranolol are being trialed to both facilitate and diminish the pathological experience of emotional arousal (the affective response) coupled to troublesome memories. In charting the development of this field, this essay aims to focus on and demonstrate how propranolol has revolutionized the treatment of PTSD and offers hope to a great percentage of critical care survivors who remain haunted by their experiences. It argues against the use of propranolol in the immediate aftermath of trauma, especially due to the incompatibility of this method with ICU treatment. Instead, it should be incorporated into and offered as part of trauma-focused psychological therapy in an extended psychological follow-up of discharged ICU patients. It suggests that, although caution is prudent and there is still much to be learnt about the benefits propranolol may have, the rapid uptake of new techniques for reducing PTSD after intensive care is necessary to maximize the quality of care given to patients.

## What is post-traumatic stress disorder?

The psychiatric diagnostic manual International Classification of Disease 10 (ICD-10) defines PTSD as ‘a delayed and/or protracted response to a stressful event or situation of an exceptionally threatening or catastrophic nature, which is likely to cause pervasive distress in almost anyone’ [4]. In 2013, the *Diagnostic and Statistical Manual of Mental Disorders* (DSM) V criteria describe four instead of three (DSM-IV) diagnostic clusters of symptoms (Table 1) [5,6]. These are characterized by the ‘persistent re-experiencing of the trauma’ , ‘avoidance of and/or numbing to stimuli associated with the trauma’ , ‘physiological arousal’ , and now also ‘negative cognitions and mood’ [4]. Few differences exist between the DSM-V and ICD-10 criteria; the latter retain the original three symptom clusters like DSM-IV, define the onset at less than 6 months, and do not specify a functional criterion [4,6]. PTSD is a debilitating anxiety disorder that is associated with both physical and psychiatric co-morbidities, a reduction in quality of life, and economic burden [7]. Approximately 80% of patients have at least one psychiatric co-morbidity, commonly including depression, alcohol and drug abuse, and other anxiety disorders [8]. Arthritis and cardiovascular and pulmonary diseases are common physical co-morbidities [9,10]. Worldwide, PTSD has a lifetime prevalence of approximately 7% [11].

**Table 1:** **Criteria for post-traumatic stress disorder in the** ***Diagnostic and Statistical Manual of Mental Disorders*** **, fifth edition**

**DSM-V - Diagnostic criteria 309.81 (F43.10)**	
**Post-traumatic stress disorder**	
A. Exposure to actual or threatened death, serious injury, or sexual violence in one (or more) of the following ways:	Directly experiencing the traumatic event(s)
	Witnessing, in person, the event(s) as it occurred to others
	Learning that the traumatic event(s) occurred to a close family member or close friend. In cases of actual or threatened death of a family member or friend, the event(s) must have been violent or accidental.
	Experiencing repeated or extreme exposure to aversive details of the traumatic event(s) (for example, first responders collecting human remains)
B. Presence of one (or more) of the following intrusion symptoms associated with the traumatic event(s), beginning after the traumatic event(s) occurred:	Recurrent, involuntary, and intrusive distressing memories of the traumatic event(s)
	Recurrent distressing dreams in which the content and/or effect of the dream are related to the traumatic event(s)
	Dissociative reactions (for example, flashbacks) in which the individual feels or acts as if the traumatic event(s) were recurring. (Such reactions may occur on a continuum, with the most extreme expression being a complete loss of awareness of present surroundings.)
	Intense or prolonged psychological distress at exposure to internal or external cues that symbolize or resemble an aspect of the traumatic event(s)
	Marked physiological reactions to internal or external cues that symbolize or resemble an aspect of the traumatic event(s)
C. Persistent avoidance of stimuli associated with the traumatic event(s), beginning after the traumatic event(s) occurred, as evidenced by one or both of the following:	Avoidance of or efforts to avoid distressing memories, thoughts, or feelings about or closely associated with the traumatic event(s)
	Avoidance of or efforts to avoid external reminders (people, places, conversations, activities, objects, situations) that arouse distressing memories, thoughts, or feelings about or closely associated with the traumatic event(s)
D. Negative alterations in cognitions and mood associated with the traumatic event(s), beginning or worsening after the traumatic event(s) occurred, as evidenced by two (or more) of the following:	Inability to remember an important aspect of the traumatic event(s) (typically due to dissociative amnesia and not to other factors such as head injury, alcohol, or drugs)
	Persistent and exaggerated negative beliefs or expectations about oneself, others, or the world (for example, ‘I am bad’, ‘No one can be trusted’, ‘The world is completely dangerous’, ‘My whole nervous system is permanently ruined’)
	Persistent, distorted cognitions about the cause or consequences of the traumatic event(s) that lead the individual to blame himself/herself or others
	Persistent negative emotional state (for example, fear, anger, guilt, or shame)
	Markedly diminished interest or participation in significant activities
	Feelings of detachment or estrangement from others
	Persistent inability to experience positive emotions (for example, inability to experience happiness, satisfaction, or loving feelings)
E. Marked alterations in arousal and reactivity associated with the traumatic event(s), beginning or worsening after the traumatic event(s) occurred, as evidenced by two (or more) of the following:	Irritable behavior and angry outbursts (with little or no provocation) typically expressed as verbal or physical aggression toward people or objects
	Reckless or self-destructive behavior
	Hyper-vigilance
	Exaggerated startle response
	Problems with concentration
	Sleep disturbance (for example, difficulty falling or staying asleep or restless sleep)
F. Duration of the disturbance (criteria B, C, D, and E) is more than 1 month	
G. The disturbance causes clinically significant distress or impairment in social, occupational, or other important areas of functioning	
H. The disturbance is not attributable to the physiological effects of a substance (for example, medication, alcohol) or another medical condition	

These criteria apply to adults, adolescents, and children older than 6 years. The *Diagnostic and Statistical Manual of Mental Disorders*, fifth edition (DSM-V), is produced by the American Psychiatric Association.

In the UK, all patients with PTSD should be offered a course of psychological treatment, either trauma-focused cognitive behavior therapy (typically involving exposure therapy, cognitive restructuring, and stress inoculation training) or eye movement desensitization and reprocessing [12]. When PTSD results from a single event, typically 8 to 12 weekly sessions are indicated; for more complex causes (for example, chronic disability and multiple events), many more sessions may be required. Typically, therapy is given in an outpatient setting; however, the role for liaison psychiatry and residential trauma treatment centers is rapidly growing [13]. Approximately 50% can achieve remission [7]. Pharmacologically, selective serotonin reuptake inhibitors (SSRIs) are considered the first-line drug treatment, to which about 60% of patients respond [7]. In the US, the SSRIs paroxetine and sertraline are approved for treating adults, whereas in the UK, only paroxetine (the only drug with a current UK product license for PTSD) and the noradrenergic and specific serotonergic antidepressant mirtazapine are recommended for the general treatment of PTSD [12,14]. The use of benzodiazepines should be primarily for short-term use when sleep is a major problem. Less commonly, amitriptyline (a tricyclic antidepressant) and phenelzine (a monoamine oxidase inhibitor) are indicated for prescription by mental health specialists [12]. Primarily prescribed as an anti-depressant, these ‘mood-altering’ drugs may not be tackling the psychopathology of PTSD in itself. To date, the medical community has not reached a consensus on a ‘gold standard’ treatment strategy. New strategies are desperately needed to help treat this increasingly recognized condition; it is hoped that treatments such as propranolol will address this need.

## Post-traumatic stress disorder after the intensive care unit

Even with conservative estimates, the current literature highlights how PTSD is a common side effect of ICU treatment. Alongside depression, it is estimated that over half the patients leaving the ICU have a psychological morbidity due to their experiences [15]. The reported prevalence of PTSD in critical care is highly variable, ranging from 5% to 64% [1,16]. A hindrance in measuring the prevalence of PTSD among ICU patients is a lack of consistency in assessment methodology (for example, differing patient populations and timing of assessments) [1]. Better evidence will help contribute to the case for the development of psychological follow-up and rehabilitation (including for PTSD) after ICU discharge.

The careful analysis of data quality is vital in assessing the prevalence of PTSD. For example, the three highest prevalences (>50%) cited by Jackson and colleagues [16] in their systematic review occurred among heterogeneous control subpopulations with sample sizes of between 11 and 27 patients. The timing of analysis (PTSD symptomatology is often time-limited) is also important and is often overlooked during data comparisons. Kapfhammer and colleagues [17] showed that PTSD prevalence drops from 43.5% at hospital discharge to 23.9% after an average of 8 years. Lastly, the tools of assessment need standardization. To demonstrate this, Jones and colleagues [18] reported that, of 102 patients, 51% had probable PTSD at a 6-month follow-up. (That study was described as the highest-quality study by Jackson and colleagues [16], a randomized control trial, and was rated according to the Oxford Centre for Evidence-based Medicine guidelines, with gradations of quality from 1a to 5 [19].) To assess PTSD-related symptoms, they used a score of more than 19 on the Impact of Event scale (IES), developed by Horowitz in 1979. However, on the original scale [20], a score of more than 19 qualified as only a non-diagnostic ‘impact event’ (‘you may be affected’). This is well below the score of at least 35 as the ideal cutoff for a probable diagnosis. Using this threshold, Myhren and colleagues [21] reported that 27% of 194 patients had symptomatic PTSD after 1 year, irrespective of physiological diagnosis. Likewise, Peris and colleagues [22] report a 57% prevalence of PTSD among ICU discharged trauma patients; a threshold score (defined by the Impact of Event Scale-Revised, or IES-R) of at least 33 was used.

A large degree of variation exists in the reported prevalence of PTSD, even while using the same diagnostic assessment tool (for example, 5% to 35% with the IES, 22.5% to 31% with the IES-R, 12.5% to 63.6% with the Post-traumatic stress syndrome 10-question inventory, 52.8% with the Davidson trauma scale, and 14% with the trauma screening questionnaire [1]). It highlights the lack of a ‘gold standard’ in assessing PTSD after ICU discharge. As intensive care patients are heterogeneous (for example, in terms of their underlying pathophysiology and duration of stay), the development of screening tools will be an important step toward identifying those who are in need of referral to specialist psychological services [23]. For example, the Post-traumatic Stress Diagnostic Scale, optimally used at 2 months after discharge, has both a high sensitivity (86%) and specificity (97%) [24]. Such tools necessitate the adoption of rigorous post-discharge follow-up, especially due to the poor predictability of which type of ICU experience will precipitate the development of PTSD. For example, the severity of the patient’s illness is not a risk factor, although the length of stay can increase the frequency of PTSD symptoms [25,26]. Nevertheless, patients admitted to the ICU, compared with other hospital patients, are at a significantly greater risk of developing PTSD [26,27]. In addition, premorbid psychopathology, prolonged sedation, and delusional memories of their experiences are risk factors after discharge [25,28-31]. PTSD is clearly a common occurrence in patients surviving the ICU and deserves the incorporation of the latest research to inform its best management.

## Why does post-traumatic stress disorder develop after the intensive care unit?

Although the physiological toll on patients admitted to the ICU is often extreme, there is no clear explanation why these events are often perceived as so psychologically traumatic. The greater risk of developing PTSD after the ICU is often irrespective of preceding clinical events (although these patients are undoubtedly at an increased risk because they are more likely to have lived through a traumatic accident) [32]. ICU admission is often associated with an increased proportion of fragmentary memories and a high proportion of delusional memories, thought to be precipitants of PTSD-related symptoms [25,28]. In addition, the extent of the physiological stress experienced in the ICU may cause pathological changes in the hypothalamic-pituitary-adrenal (HPA) axis (coordinates the stress response) which predisposes patients to PTSD [33]. Disturbed and disrupted sleep is extremely common in the ICU [34], a pattern that has been associated with the development of PTSD [35]. The role of sleep in encoding, refining, and enhancing memories (for recollection) from the day before is slowly being delineated [36-38]. As the nature of this process requires memories to be reactivated, they become vulnerable to disruption (‘labile’), which may underlie the memory difficulties of many ICU patients. However, although sleep disturbance is a common symptom of PTSD, it may be only a correlative association not causative. Regardless of the cause, the ICU package of care must incorporate psychological rehabilitation in addition to its ability to restore physical health.

## Where we are now: managing post-traumatic stress disorder after the intensive care unit

The best practice for reducing the prevalence of post-ICU PTSD has yet to be defined. Specific psychological interventions have been trialed with some success. This represents the vanguard of an increasing role for psychiatry liaison services in the ICU. Peris and colleagues [22] employed early 24-hour psychological support for trauma patients during admission and demonstrated over a 50% reduction in PTSD. After recovery of consciousness, patients received approximately five or six interventions from clinical psychologists, including education, counseling, and stress management. However, other studies using nurse-led follow-up programs [29] and self-help rehabilitation manuals [18] have shown little benefit. Diary interventions, aimed at aiding therapeutic recollection of the ICU, have been shown to reduce the incidence of PTSD (5%) compared with controls (13%) [39,40]. These studies represent a promising start in addressing the psychiatric complications that are common among patients discharged from the ICU. Yet as our understanding of PTSD has grown, intensivists must strive to incorporate an evolving neurochemical understanding of this disorder into their patient management.

## The neurochemical basis of post-traumatic stress disorder

PTSD occurs when a terrifying event overstimulates a sympathetic ‘fight-or-flight’ hormonal response, which then strengthens its memory (the conditioned fear) and later manifests as an over-reactive fearful response to reminders of the event [41,42]. An over-reactive limbic system, which includes our amygdala (our fear center), is hypothesized to mediate the aberrant conditioning process in which a hormonal response is fixed to a memory [43]. Alterations to the HPA axis have also been implicated [44]. For example, heightened noradrenaline concentrations are found in the cerebrospinal fluid of patients with PTSD [45]. Dysfunction is also found in the ability of the hippocampus (the primary site for memory storage) to regulate the recollection of fearful events. This may underlie the recurrent nature of the symptoms, especially to seemingly neutral stimuli [46].

Although the exact process of dysfunction is unclear, a combination of a low threshold for ‘fear stimuli’ stimulating recall coupled with an over-reactive emotional ‘fight-or-flight’ response to them is likely to be involved. By understanding how this link is formed in the process of memory formation, new therapies which aim to uncouple the debilitating affective response from a recollection of trauma can be developed. Propranolol is a novel pharmacological aid that may offer hope to a growing need for effective post-ICU psychological rehabilitation. Other than this, other pharmacological candidates include histone deacetylases, brain-derived neurotrophic factor, cannabinoids, glucocorticoids, mifepristone, and valproic acid. Though beyond the scope of this review, a thorough analysis of their use is provided by Fitzgerald and colleagues [47] (2014).

## Malleable memories

In 1979, Elizabeth Loftus asserted that memory was malleable and not hard-wired into the brain [48]. She demonstrated that recollection can be supplemented or even altered by psychological manipulation. Although the process of encoding, consolidating, and storing was accepted (the process by which a thought is fixed in memory), the idea of retrieving a memory (that is, ‘reactivating’ it), making it again liable to change before being stored again once more (reconsolidation), would fundamentally change the field.

Memories are first stored in our short-term memory. This state can easily be interfered with; a distraction can often lead to a disruption of the memory-encoding phase and as a result the memory can be forgotten [36]. Over the next hours, structural changes occur (for example, in the hippocampus) which permit long-term memory: long-term potentiation (LTP). In the late phase of LTP, neurons synthesize proteins to provide new and lasting synaptic changes. Blocking glutamate activation of N-methyl-D-aspartate receptors pharmacologically interferes with LTP and memory storage [49]. A day later, the name you learnt yesterday will be resistant to the same distraction - it is consolidated.

In a landmark study, Walker and colleagues [37] provided evidence that re-consolidation could occur for human memories. Using a finger-tapping task (learning a sequence of taps) they first demonstrated that a period of sleep can enhance performance (recollection). Learning a second sequence after learning a first will only interfere with recall if it is learnt immediately rather than 6 hours after, implying that consolidation has occurred during this time. However, most importantly, they showed that this consolidation could be reversed. Brief rehearsal of the first sequence learnt a day before, immediately before learning a second, significantly decreased performance of the first on the third day. This implies that the reactivation of the memory returned it to a labile state, making it susceptible to interference again. This finding and others like it underlie attempts to clinically exploit the phenomenon of reconsolidation, debilitating the affective response to harmful memories that pervade psychiatric diseases like PTSD [36].

## Decoupling response from fear: a role for propranolol?

In searching for a way to pharmacologically block memories, Einarsson and Nader [50] showed how the protein-synthesis inhibitor anisomycin injected into the anterior cingulate cortex of a rat could prevent the reconsolidation of an affective response to a feared stimulus. Rats were conditioned to associate a tone with a painful electric foot shock; when the tone was played in the absence of the foot shock, the rats still displayed a fear response. The next day (after consolidation), the tone was played again (reactivation) with an immediate injection of anisomycin. This successfully blocked the fear reaction 2 weeks later [50]; however, anisomycin given without reactivation of the memory meant no effect was observed [51]. Einarsson and Nader [50] concluded that anisomycin was able to block the process of reconsolidation; the original response linked to the memory is forgotten. Others have suggested that its effect is instead one of a temporary amnesia, not inhibition of reconsolidation [52]. Current ambiguity with regard to the mechanism of reconsolidation and differences in study methodology make such results hard to compare. Yet these observations offer hope that the phenomenon of reconsolidation could be exploited therapeutically.

The known pro-apoptotic effects of anisomycin have prevented its use in humans [51], so other agents were sought after. Previous research had suggested that the beta adrenergic receptor antagonist propranolol had the capacity to impair declarative memory in humans [53] and the process of memory consolidation and reconsolidation in rats [54,55]. Paving the way for human trials, Debiec and Ledoux [56] demonstrated that both intra-amygdala and systemic infusions of propranolol were able to interfere with previously learnt memories during reconsolidation in rats (although not the process of consolidation). Propranolol is thought to antagonize the transmission of stress hormones, which modulate the activation and memory-encoding function of the amygdala [57]. A hypothesis formed that propranolol may not only help extinguish memories during reconsolidation but also prevent the immediate link between a traumatic memory and a hormonal response ever being formed (‘over-consolidation’) [58].

Studies attempting to inhibit over-consolidation have had poor results. Whereas some small studies have demonstrated that physiological arousal 3 months after a trauma can be significantly reduced with a 10-day course of propranolol [59,60], others have failed to demonstrate an effect versus placebo in burden veterans [61], among trauma patients [62], or using high-dose therapy [63] (240 mg/day for 19 days). A crucial difference separated these studies from the original protocols in murine models: the reactivation of traumatic memories was not done alongside propranolol. Focusing psychopharmacological treatment on over-consolidation ignores the principal finding of Debiec and Ledoux [56] in their rat model: that propranolol was effective at suppressing the affective response coupled to memories after reconsolidation but not consolidation.

The theoretical requirement that propranolol, and drugs like it, can only alter the process of reconsolidation is not a hindrance for its proposed use in post-ICU PTSD. Discharged ICU patients are often in a precarious physiological state, so an immediate dose of an adrenergic antagonist may dangerously destabilize a patient (or simply supplement a standing prescription). In this sense, a psychopharmacological intervention temporally distant from discharge would be most suitable for post-ICU patients. As the field has advanced, more studies have investigated the modulation of memories by propranolol after their reactivation during the process of reconsolidation.

## The reconsolidation effect

With the idea that memories must be reactivated before they can become liable to change, Brunet and colleagues [41] asked patients with chronic PTSD to describe their trauma in a script preparation session. They were then immediately given a single dose of ‘post-retrieval’ propranolol. A week later, these patients had significantly smaller physiological (that is, ‘fight-or-flight’) responses to traumatic cues. The authors hypothesized that if the previously coupled state of sympathetic arousal was inhibited pharmacologically during the process of reconsolidating reactivated memories, the newly re-stored memory would be permanently dissociated from it [41]. In addition, propranolol has been shown to specifically diminish the accuracy of the remembering of negative images after reactivation, while leaving the recollection of neutral images unchanged [64].

Further studies using functional magnetic resonance imaging have shown that propranolol given after memory reactivation can reduce the recognition of emotional pictures [65]. Neither propranolol without reactivation nor reactivation alone had any effect on subsequent memory. Only when a picture was successfully recognized was there significantly increased activity in the amygdala and hippocampus in those who experienced emotional memory impairment. This may suggest memories that have the strongest encoding could survive being impaired by propranolol [66]. Participants were also scanned during reactivation showing no immediate effect on brain activity from propranolol, suggesting that it does not exert a direct effect on the process of reactivation but in the hours after (in the ‘reconsolidation window’).

The effect of propranolol has also been shown in response to fear conditioning [67]. The response to a loud noise, coupled to exposure of a fear-relevant stimuli (for example, pictures of spiders), was measured by the eye-blink startle reflex. Drug administration before reactivation completely eliminated the behavioral response to the fear memory 24 hours later. In addition, retrieval techniques were unable to reinstate the fear response. The authors and others suggest that propranolol somehow blocks the protein synthesis in the amygdala which strengthens the link between the arousal response and the fear memory. The subsequent interference results in both aspects of heightened fear and arousal being either partially erased or unavailable for retrieval in such a distressing and vivid way [64,67,68].

## Refining the propranolol protocol

The work of Brunet and colleagues [41] in 2008 paved the way for further open-label trials in patients with diagnosed PTSD, in an attempt to refine the treatment protocol. For each trial, they used six sessions in which two doses of propranolol were given: one short-acting dose (40 mg) immediately before traumatic memory reactivation and another long-acting dose (60 mg) 90 minutes after [69]. A 71% reduction in the number of participants no longer meeting the criteria for PTSD was achieved. When comparing patients who all developed PTSD following the 2001 Toulouse industrial disaster, 86% of treated patients lost their diagnosis, compared with 8% in the control group. Propranolol not only shows promise in its own right but shows how memory can be therapeutically altered.

Concurrently, in a similar protocol-refinement exercise (with only one session but the same pre-reactivation and long-acting post-treatment dose), a 2008–2010 case series reported by Menzies [70] showed a 91.7% response rate and 43% to 58% reduction in PTSD self-rating measures. The mean score on the patient global improvement scale was 8.4 out of 10, equating to ‘markedly improved’. These benefits have been sustained for up to 2.5 years. These studies represent a new and exciting proof-of-principle for future work. Propranolol seems to reduce both the intensity and frequency of traumatic memories and the associated emotional distress [70]. Traumatic memories are uncoupled from their affective response, with a degree of associated amnesia.

Propranolol-mediated therapies have shown a rapid and prolonged reduction in PTSD symptoms and these qualities are especially important in the context of the ICU. Often, ICU patients have multiple physical co-morbidities which may be exacerbated, or their treatment disrupted, by such a pervasive psychological condition. In this sense, this new development represents an exciting new tool in treating PTSD as part of an under-developed psychological follow-up of ICU survivors. However, the potential harm of any new treatment must be thoroughly investigated. For example, the exclusion criteria used by Brunet and colleagues [41] (2008) may represent a high proportion of ICU patients, including the concurrent use of a beta antagonist, hypotension, asthma, heart failure, certain arrhythmias, and insulin-requiring diabetes. Although this may limit the use of propranolol, especially in multi-morbid and older patients, it does not discount its potential benefit for many others. Therefore, future trials should focus not only on whether it is an effective treatment but for whom it can be effective and safely administered. Ethical concerns over the use of propranolol cannot levy the charge of altering ‘who we are’, our identity, because the nature of our ‘true’ experiences is constantly changing despite its influence [71]. Both psychological and pharmacological strategies modify memories in the mind. As long as a balanced approach to treatment is maintained, the risk that propranolol may over-medicalize or undermine coping strategies already employed by ICU patients is low.

## Conclusion: psychopharmacology after the intensive care unit

Increasingly, the realization that the role of intensive care specialists may extend beyond the ICU is changing clinical practice. Mediating and incorporating an interdisciplinary approach to care are vital in this respect; psychiatry is no exception. PTSD is often associated with emotional memories which are distressing, potent, and idiosyncratic. All three terms are also true of a patient’s experience in the ICU; it is a traumatic experience. As traumatic memories are re-stored, propranolol acts to dissociate the state of sympathetic arousal from their recollection. Studies, largely in young healthy adults, have shown its ability to reduce the recall and response to negative or fear-inducing stimuli. Future research into propranolol, including its use in the ICU, must diversify and broaden studied populations to improve their ecological validity and examine long-lasting effects in clinical patients. Well-conducted large randomized trials will be an important aspect of improving the evidence base, as much is still unknown about the benefits of propranolol. Only after this should propranolol be considered for use in treating PTSD after the ICU. In a broader sense, a program of extended follow-up after discharge, aided by screening tools and liaison psychiatry, should be incorporated into standard ICU treatment. Trauma-focused psychological (for example, cognitive behavioral therapy) and pharmacological (for example, paroxetine) therapies should be employed alongside the latest research endorsing propranolol-facilitated affective dissociation from traumatic memories. As this field advances, intensivists and psychiatrists alike should collaborate in using the latest psychopharmacology to treat their patients, combating the psychological consequences of experiencing the extremes of physiological existence.

## Abbreviations

DSM: *Diagnostic and Statistical Manual of Mental Disorders*
HPA: Hypothalamic-pituitary-adrenal
ICD-10: International Classification of Disease 10
IES: Impact of Event Scale
IES-R: Impact of Event Scale-Revised
LTP: Long-term potentiation
PTSD: Post-traumatic stress disorder
SSRI: Selective serotonin reuptake inhibitor
